# Tim-1 Deficiency Aggravates High-Fat Diet-Induced Steatohepatitis in Mice

**DOI:** 10.3389/fimmu.2021.747794

**Published:** 2021-10-05

**Authors:** Jasmine George, Yuanyuan Zhang, Jacob Sloan, Joya M. Sims, John D. Imig, Xueying Zhao

**Affiliations:** ^1^Department of Physiology, Morehouse School of Medicine, Atlanta, GA, United States; ^2^Drug Discovery Center, Medical College of Wisconsin, Milwaukee, WI, United States

**Keywords:** non-alcoholic steatohepatitis, high-fat diet, lipid metabolism, inflammation, Tim-1

## Abstract

Non-alcoholic fatty liver disease (NAFLD)/non-alcoholic steatohepatitis (NASH) is commonly associated with obesity and characterized by excessive lipid accumulation and liver inflammation. The T cell immunoglobulin and mucin domain 1 (Tim-1), also known as hepatitis A virus cellular receptor 1 (Havcr-1) and kidney injury molecule 1 (Kim-1), has been shown to affect innate immunity-driven proinflammatory cascade in liver ischemia-reperfusion injury. However, its contribution to obesity-related NAFLD/NASH remains unknown. Thus, this study was designed to evaluate the role of Tim-1 in obesity-related liver inflammation and injury in wild-type (WT) and Tim-1-deficient (Tim-1^-/-^) C57BL/6J mice fed a high-fat diet (HFD) for 5-6 months. HFD feeding induced steatosis and upregulated Tim-1 gene expression in the liver of WT mice. Surprisingly, Tim-1^-/-^ mice on HFD diet exhibited an exacerbation of hepatic steatosis, accompanied with an elevation of protein levels of fatty acid translocase CD36 and sterol regulatory element binding protein 1 (SREBP1). Tim-1 deficiency also enhanced HFD-induced liver inflammation and injury, as evidenced by augmented increase in hepatic expression of pro-inflammatory factor lipocalin 2 and elevated serum alanine transaminase (ALT). In addition, gene expression of type I, III and IV collagens and liver fibrosis were greatly enhanced in HFD Tim-1^-/-^ mice compared with HFD WT mice. HFD-induced hepatic expression of YM-1, a specific mouse M2 macrophage marker, was further upregulated by deletion of Tim-1. Together, these results show that Tim-1 deficiency aggravates the effects of HFD diet on lipid accumulation and liver fibrosis, most likely through enhanced infiltration and activation of inflammatory cells.

## Introduction

Non-alcoholic fatty liver disease (NAFLD) has become the most common cause of chronic liver diseases in western countries (1). The prevalence of NAFLD in the U.S. adult population is approximately 25%, and about one-fourth of the patients with NAFLD progress to nonalcoholic steatohepatitis (NASH) (2). Through histological examinations, NASH is characterized by steatosis, hepatocyte ballooning, lobular inflammation, and varying degrees of liver fibrosis, which can lead to scarring of the liver (3). NASH has become a major health concern; however, there are no FDA-approved drugs for the treatment of this disease. Thus, improving our understanding of the functions of genes/mediators that contribute to the susceptibility to and severity of NAFLD/NASH is of great consequence to both the treatment and prevention of chronic liver disease.

The liver is recognized as an innate immunity organ and the hallmark of NASH is markedly enhanced infiltration of various immune cells, including hepatic macrophages (liver-resident Kupffer cells and recruited monocyte-derived macrophages), neutrophils, monocytes, and natural killer T cells (4–6). Given the functional role of hepatic macrophages as a master regulator of immune homeostasis and a pivotal coordinator of liver inflammation, it has been postulated that macrophage activation and polarization are involved in the progression of NAFLD to NASH. Nevertheless, it remains largely unknown how M1 *versus* M2 macrophages contribute to liver inflammation and fibrosis progression in advanced NASH.

T cell immunoglobulin and mucin domain-containing molecule 1 (Tim-1), also known as hepatitis A virus cellular receptor 1 (Havcr-1) and kidney injury molecule 1 (Kim-1), was first identified in kidney cells of African green monkeys in 1996 (7). Tim-1 protein, identified in activated T cells, B cells and dendritic cells, functions as co-stimulators and co-inhibitors of immune responses. For example, studies using blocking or activating antibodies have shown that the interaction between Tim-1 and ligand can enhance the activation of T cells and increase the production of Th2 type cytokines while blocking this interaction can greatly inhibit the activity of Th2 cells, thus regulating the immune response mediated by Th2 cells (8–15). Notably, a study performed in *in vivo* allergic airway disease revealed enhanced production of the Th2 cytokines (e.g., IL-4, IL-5, and IL-13) and inflammatory responses in the absence of Tim-1, suggesting that its primary role is to dampen, rather than promote, Th2-type immune responses (16). This concept is also supported by the findings that blockade of Tim-1 in low-density lipoprotein receptor (ldlr)-deficient mice aggravates atherosclerosis, which is likely related to the change in Th1/Th2 balance and reduced circulating regulatory T cells (17). Interestingly, Tim-1 signaling has been confirmed to be necessary for liver ischemia-reperfusion injury *via* increasing T cell, neutrophil, and macrophage sequestration (18, 19). High Tim-1 expression was observed in liver graft during ischemia-reperfusion injury development, and inhibition of Tim-1 abolished neutrophil and macrophage infiltration/activation in liver transplantation (18, 20) and subsequently ameliorated hepatocellular damage and improved liver function (18). Based on these observations, we hypothesized that Tim-1 plays a role in diet-induced NAFLD/NASH *via* its modulation of the inflammatory response to metabolic stress.

In this study, we generated mice deficient in Tim-1 and evaluated their responses to chronic high-fat diet (HFD) treatment. We found that the Tim-1 gene was significantly upregulated in HFD-fed wild-type (WT) livers. Tim-1 deficiency led to an exacerbation of HFD-induced steatosis concomitant with enhanced liver infiltration of neutrophils as well as macrophage activation and M2 polarization.

## Materials And Methods

### Animal Experiments

The Tim-1/Havcr-1/Kim-1 knockout (Tim-1^-/-^) mouse strain was generated using CRISPR/Cas9 technology on a C57BL/6J genetic background by the Gene Edit Biolab (Atlanta, GA, USA). Tim-1^-/-^ mice were produced through heterozygous breeding. Tail samples were collected and genotyped. The animals were housed in a temperature-controlled room with 12 h light/dark regimen. All animal experiments were approved by the Institutional Animal Care and Use Committee at the Morehouse School of Medicine and were performed according to strict government and international guidelines on animal experimentation.

Male WT and Tim-1^-/-^ mice at the age of 6 - 8 weeks were fed with standard chow or HFD diet for 5 - 6 months. The HFD, containing 42% fat, was purchased from the Envigo (TD.88137; Madison, WI, USA). Mouse body weight was measured every week, and blood glucose was monitored biweekly. The mice were fasted for 5-6 hours before euthanasia. Blood was collected by heart puncture. Liver and kidney tissues were harvested and stored in 4% paraformaldehyde or quickly frozen in liquid nitrogen and then stored at -80°C until further use.

### Biochemical Analysis

Blood samples were clotted at room temperature for 2 hours and then centrifuged at 1200xg for 20 minutes. Serum samples were assayed immediately or split and stored at -80°C, avoiding repeated freeze-thaw cycles. Serum insulin concentration was measured using a kit from Thermo Fisher Scientific (Carlsbad, CA, USA). The serum lipid levels of cholesterol and triglyceride were measured to evaluate the relative lipid content changes *via* commercially available kits provided by Wako Chemicals USA Inc. (Richmond, VA, USA). Serum alanine aminotransferase (ALT) concentration was determined to evaluate liver function using an ALT activity assay kit (Thermo Fisher Scientific).

### Quantitative Reverse Transcription PCR Analysis

Total RNA from mouse liver and kidney cortex was extracted using TRIzol (Invitrogen, Carlsbad, CA, USA) according to the manufacturer’s instructions. Quantitative reverse transcription PCR (RT-qPCR) was performed using SYBR Green PCR Master Mix (Applied Biosystems, Foster City, CA, USA) and primers for mouse genes (**Table 1**). GAPDH was used as endogenous control. Each sample was run in triplicate, and the comparative threshold cycle (C_t_) method was used to quantify fold increase (2^-ΔΔCt^) compared with controls.

**Table 1:** Primer sequences for qPCR.

Mouse genes	Forward (5’-3’)	Reverse (5’-3’)
Tim-1	AAACCAGAGATTCCCACACG	GTCGTGGGTCTTCCTGTAGC
Lcn2	CCATCTATGAGCTACAAGAGAACAAT	TCTGATCCAGTAGCGACAGC
Col1a1	CCAAGAAGACATCCCTGAAGT	GTGGCAGATACAGATCAAGCA
Col3a1	CGTAGATGAATTGGGATGCA	ACATGGTTCTGGCTTCCAG
Col4a1	GCTGCCTGCGTAAGTTCAG	CGTGGACAGCCAGTAAGAGT
Fn1	TTTGACAATGGGAAGCACTATC	CAAACCAGGGCGTTGC
CD11c	CTGGATAGCCTTTCTTCTGCTG	GCACACTGTGTCCGAACTCA
TNF-α	ACGGCATGGATCTCAAAGAC	AGATAGCAAATCGGCTGACG
CD206	CATGGATGTTGATGGCTACTGGAG	GTCTGTTCTGACTCTGGACACTTG
YM-1/Chil3	AGAGTGCTGATCTCAATGTGG	GGGCACCAATTCCAGTCTTAG
TGF-β1	TGCTAATGGTGGACCGCAA	CACTGCTTCCCGAATGTCTGA
IL-10	ATGCTCCTAGAGCTGCGGACT	CCTGCATTAAGGAGTCGGTTAG
GAPDH	CATCACTGCCACCCAGAAGACTG	ATGCCAGTGAGCTTCCCGTTCAG

### Light Microscopy of Liver Sections

Ten percent formalin-fixed paraffin sections (5 µm) were stained with hematoxylin and eosin (H&E) for histological evaluation. Another set of paraffin-embedded liver sections were stained with Sirius Red to identify collagen. Neutral lipid accumulation was determined by oil red O (ORO) staining of frozen hepatic tissue sections, which allows detection of triglyceride and cholesterol esters. The slides were observed and imaged by the Olympus microscope. All histological analysis was conducted by two observers in a blinded fashion. Sirius Red or ORO-stained areas were quantified by ImageJ software (21).

### Western Blot Analysis

Liver tissue samples were lysed with RIPA lysis buffer (Sigma Aldrich Inc., St. Louis, MO, USA) and a protease inhibitor cocktail (Sigma-Aldrich). Protein (30 µg) samples were separated by 4-20% SDS-PAGE and transferred electrophoretically to nitrocellulose membranes (GE Healthcare, Piscataway, NJ, USA). The blots were incubated with primary antibodies for lipocalin-2 (LCN2, 1:1000, R&D, Minneapolis, MN, USA), fatty acid transport protein (FATP)2 (1:1000, Novus Biologicals, Centennial, CO, USA), FATP5 (1:1000, Novus Biologicals), CD36 (1:1000, Abcam, Cambridge, MA, USA), sterol regulatory element binding protein (SREBP)1 (1:500, Santa Cruz Biotechnology, Santa Cruz, CA, USA), Ly6G/6C (1:1000; BD Diagnostic Systems, Sparks, MD, USA), F4/80 (1:1000; Bio-Rad Laboratories Inc., Irvine, CA, USA), and GAPDH (1:6000, Sigma-Aldrich). After incubation with HRP-conjugated secondary antibody, signals were detected using enhanced chemiluminescence ECL reagents (GE Healthcare). Relative band intensity was measured densitometrically by ImageJ software with GAPDH, a housekeeping protein, as an internal control.

### Immunostaining

To examine the expression and distribution of LCN2, 5-µm cryostat liver sections were incubated with one or two primary antibodies overnight: goat anti-LCN2 (1:100; R&D) and rat anti-Ly6G/6C (a marker of neutrophils; 1:100; BD Diagnostic Systems) or goat anti-LCN2 and rat anti-F4/80 (a marker of macrophages; 1:100; Bio-Rad Laboratories Inc.). The secondary antibodies were Alexa Fluor 488-conjugated donkey anti goat or rat IgG (1:200) or Alexa Fluor 555-conjugated donkey anti-goat or rat IgG (1:200) from the Jackson ImmunoResearch Laboratories (West Grove, PA, USA). As a negative control, the sections were exposed to nonimmune IgG (in replacement of primary antibodies) with the same secondary antibodies, and no specific staining was observed. After nuclear staining with DAPI, the slides were mounted with ProLong gold antifade reagent (Thermo Fisher Scientific). The sections were observed and imaged by a Leica confocal microscope (Wetzlar, Germany).

### Statistical Analysis

Data are expressed as means ± SEM. Student’s t-test was used for comparison between two groups. Comparisons among multiple groups were performed by one-way ANOVA and Tukey *post hoc* test. Differences were considered statistically significant at *P* < 0.05.

## Results

### Tim-1 Gene Expression Was Upregulated in HFD-Fed C57BL/6J Mouse Livers

**Figure 1:**
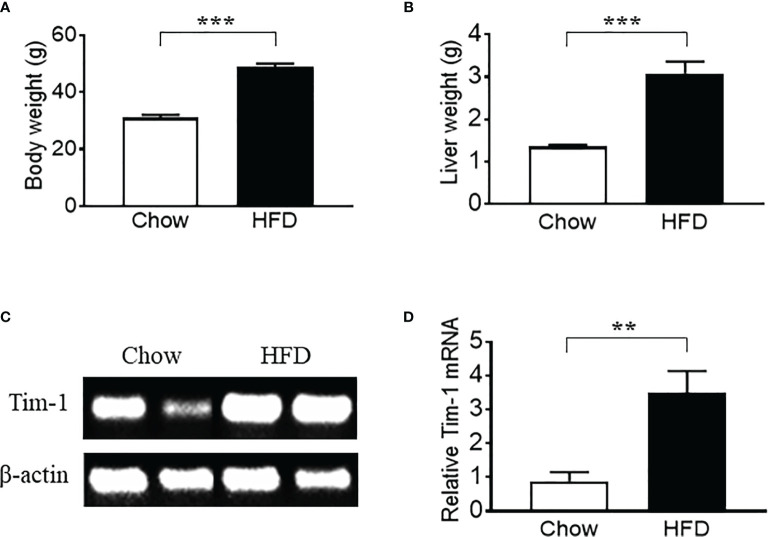
High-fat diet (HFD) feeding for 6 months led to an upregulation of hepatic Tim-1 in wild-type C57BL/6 mice. **(A, B)** Increased body weight and liver weight in HFD-fed mice compared to chow controls. **(C, D)** Representative gel and quantitative analysis after 35 cycles RT-PCR revealed an upregulation of Tim-1 gene expression in HFD mouse liver. Values are mean ± SEM. n = 5-6 mice; **P<0.01, ***P<0.001 vs. chow control group.

Previous studies have shown that hepatic expression of Tim-1 is increased in liver ischemia-reperfusion injury (18–20). Here, we examined the effect of metabolic stress on Tim-1 expression in the liver of C57BL/6J WT mice. Compared to chow control group, HFD feeding for 5-6 months significantly increased body weight (**Figure 1A**) and liver weight (**Figure 1B**). In addition, real-time qPCR analysis revealed an upregulation of hepatic Tim-1 transcript in HFD-induced obese mice compared with chow controls (**Figures 1C, D**), whereas its mRNA levels were not different in kidney tissues of chow (1.1 ± 0.2, n=5) and HFD (0.9 ± 0.1, n=4) WT mice.

### Tim-1 Deficiency Enhanced HFD-Induced Hepatic Steatosis

**Figure 2:**
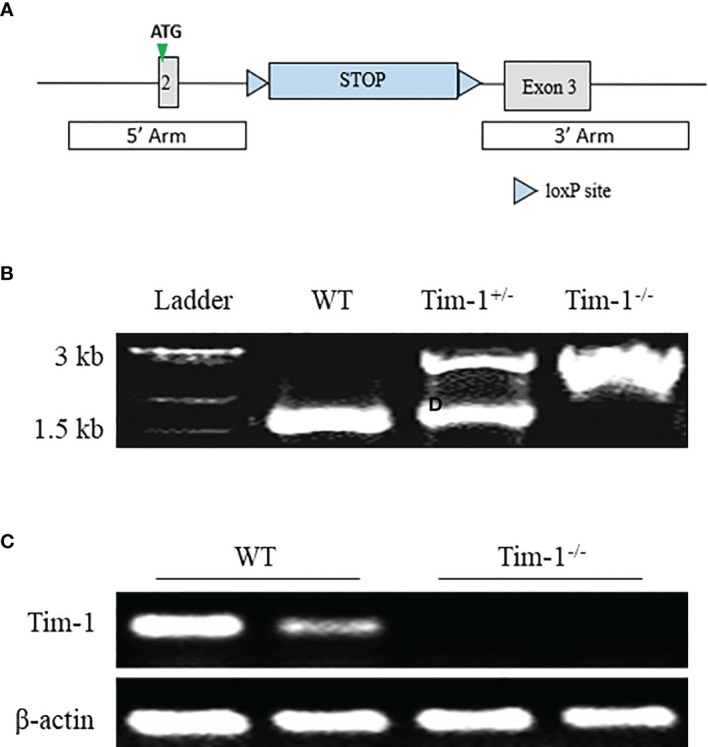
Generation of global Tim-1 knockout mice. **(A)** Schematic diagram of generation of Tim-1 Lox-Stop-Lox knock-in (Tim-1^-/-^) mice. **(B)** Genotyping PCR of tail DNA from wild-type (WT), heterozygous (Tim-1^+/-^), and homozygous (Tim-1^-/-^) mice. **(C)** Representative image of hepatic Tim-1 gene after 35 cycles RT-PCR in WT and Tim-1^-/-^ mice.

To further explore the function of Tim-1 in diet-induced hepatic steatosis, Tim-1^-/-^ mice on C57BL/6J background were generated by inserting a Lox-Stop-Lox cassette into intron 2 of Tim-1 using CRISPR/Cas9 technology (**Figures 2A, B**). As expected, Tim-1 mRNA was not detectable in the liver (**Figure 2C**) of Tim-1^-/-^ mice. The Tim-1^-/-^ mice on chow diet had normal size and displayed normal physical behavior and activity. Moreover, deletion of Tim-1 had no effects on body weight gain and blood glucose in mice fed with chow or HFD diet. Serum insulin levels were similarly increased in HFD-fed WT (5.51 ± 1.29 ng/ml, n=6) and Tim-1^-/-^ (6.58 ± 2.24 ng/ml, n=4) compared to chow-fed WT (1.73 ± 0.18 ng/ml, n=5) and Tim-1^-/-^ (2.29 ± 0.37 ng/ml, n=6) mice.

**Figure 3:**
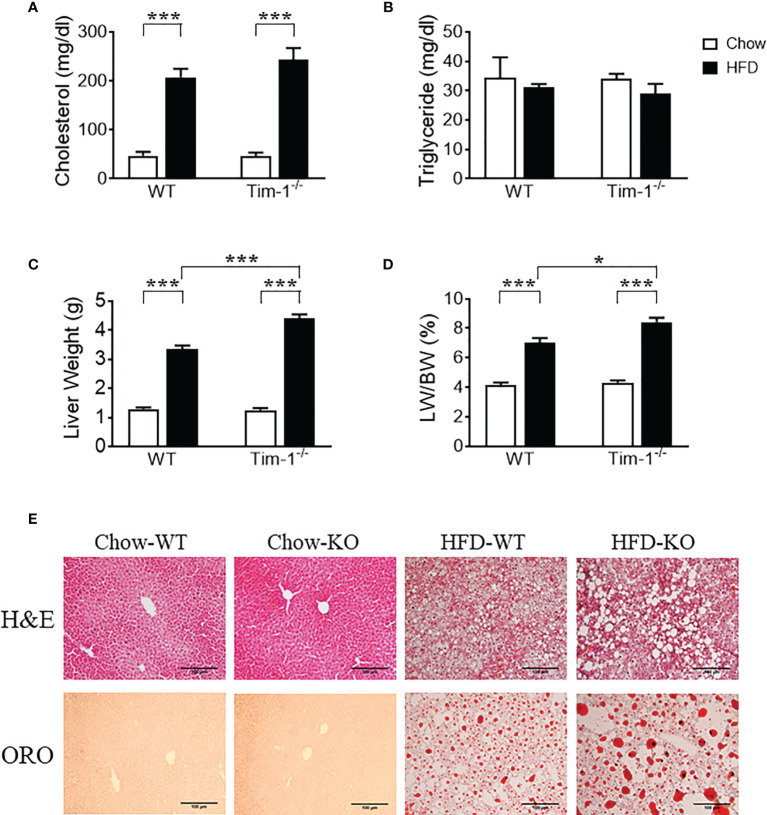
Tim-1 deficiency accelerated diet-induced hepatic steatosis. Serum levels of cholesterol **(A)** and triglyceride **(B)**, liver weight **(C)**, and liver weight to body weight (LW/BW) ratio **(D)** in chow or high-fat diet (HFD) WT and Tim-1-dificient (Tim-1^-/-^) mice. **(E)** Representative images of H&E and Oil red O (ORO) staining of WT and Tim-1-deficient (KO) livers (scale bar = 100 µm). Values are mean ± SEM. n=4-6 mice. Statistical differences were assessed by one-way ANOVA with Tukey’s multiple comparisons test; *P<0.05, ***P<0.001.

Serum triglyceride and total cholesterol levels were measured to evaluate the effect of Tim-1 deficiency on mouse lipid profile in serum. Compared to chow controls, HFD feeding led to a similar increase in cholesterol levels in WT and Tim-1^-/-^ mice (**Figure 3A**), whereas triglyceride levels were not different among all four groups (**Figure 3B**). Although Tim-1 deficiency did not alter the liver weight and liver index (liver weight to body weight ratio) in chow-fed mice, there was a greater increase in liver weight and liver index in HFD-fed Tim-1^-/-^ mice compared with HFD WT animals (**Figures 3C, D**). To further confirm the effect of Tim-1 deficiency on hepatic steatosis, we next evaluated lipid accumulation using H&E and ORO staining with liver sections. As shown in **Figure 3E**, there was no significant difference in liver histology and lipid storage between WT and Tim-1^-/-^ mice on chow diet. However, HFD-induced hepatocyte ballooning and neutral lipid accumulation were more prominent in mice lacking Tim-1. ORO-positive area averaged 5.8 ± 1.1% in HFD-fed WT livers, which was further increased to 10.2 ± 1.3% (*P*<0.05) in HFD Tim-1^-/-^ ones.

### Tim-1 Deletion Enhanced HFD-Induced Hepatic Expression of Proteins Involved in Lipid Uptake and Biogenesis

**Figure 4:**
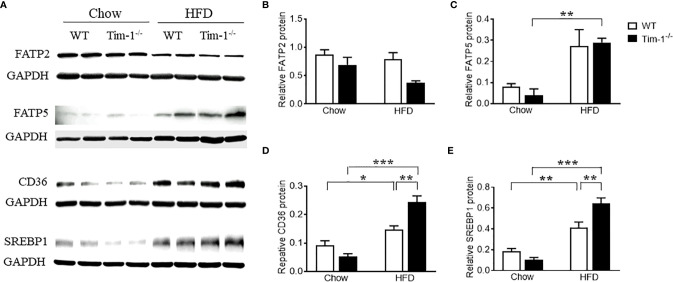
Tim-1 deficiency increased protein levels of FATP5, CD36 and SREBP1 in HFD mouse livers. **(A)** Representative Western blot images of FATP2, FATP5, CD36, and SREBP1 in the liver of chow or HFD-fed WT and Tim-1^-/-^ mice. **(B–E)** Quantitative analysis of each protein. Values are mean ± SEM. n=4-6 mice. Statistical differences were assessed by one-way ANOVA with Tukey’s multiple comparisons test; *P<0.05, **P<0.01, ***P<0.001.

To understand the mechanisms by which Tim-1 deficiency accelerates diet-induced hepatic steatosis, we next determined the proteins involved in the transport and biogenesis of fatty acids (FAs). Hepatic FA uptake is mainly mediated by SLC27A/FATPs and FA translocase CD36 (22, 23). Two members of the FATP family, FATP2 and FATP5, are robustly expressed in liver (24) and are thought to be involved in the early steps of long-chain FA uptake/activation (25, 26). Thus, we first compared the protein levels of FATP2 and FATP5 in the liver of chow or HFD-fed WT and Tim-1^-/-^ mice. As shown in (**Figures 4A, B**), there was no significant difference in hepatic FATP2 protein among the four groups. Compared to chow controls, an upregulation of FATP5 protein expression was observed in HFD Tim-1^-/-^ mice but not in HFD WT mice (**Figures 4A, C**). The FA translocase protein CD36 has been shown to accelerate FA uptake and extensive incorporation into triglycerides (27). Compared to chow controls, CD36 protein was significantly increased in liver tissues of HFD-fed WT mice, which was further enhanced by Tim-1 deletion (**Figures 4A, D**).

As a family of transcription factors involved in the biogenesis of cholesterol, FAs and triglycerides, SREBPs are implicated in the pathogenesis of NAFLD/NASH and SREBP1 is the primary subtype expressed in the liver of mice and humans (28, 29). Here, we confirmed that HFD feeding increased SREBP1 protein in WT livers (**Figures 4A** **, E**). Although Tim-1 deficiency had no effect on SREBP1 expression in the liver of chow-fed mice, HFD-induced upregulation of SREBP1 was greatly augmented by deletion of Tim-1 (*P* < 0.001 *versus* HFD WT group).

### Tim-1 Deletion Accelerated HFD-Induced Liver Injury and Inflammation

**Figure 5:**
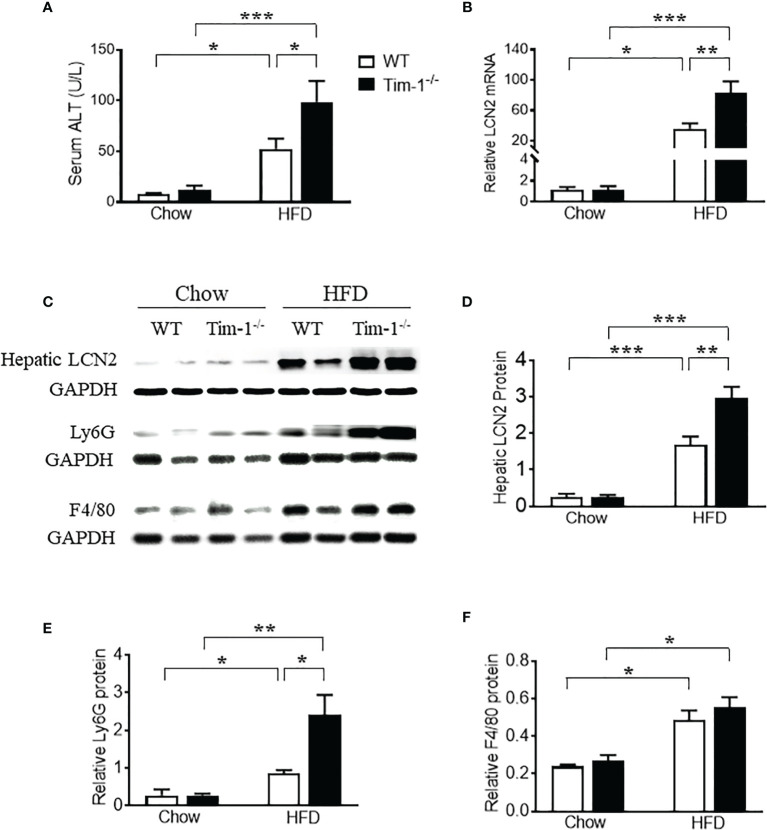
Tim-1 deficiency enhanced HFD-induced liver injury. **(A)** Serum alanine aminotransferase (ALT) concentration in chow or HFD-treated WT and Tim-1^-/-^ mice. **(B)** Real-time qPCR analysis of lipocalin 2 (LCN2) transcript in chow or HFD-fed WT and Tim-1^-/-^ mouse livers. **(C–F)** Representative Western blot image and quantitative analysis of hepatic LCN2, Ly6G/C, and F4/80 proteins. Values are mean ± SEM. n=4-6 mice. Statistical differences were assessed by one-way ANOVA with Tukey’s multiple comparisons test; *P<0.05, **P<0.01, ***P<0.001.

In accordance with the more severe hepatomegaly and NAFLD liver phenotype shown by H&E and ORO staining in HFD-fed Tim-1^-/-^ mice, HFD induced more hepatic secretion of ALT, a useful biomarker of liver injury, in mice lacking Tim-1. As shown in **Figure 5A**, HFD-fed Tim-1^-/-^ mice showed significantly higher serum ALT level compared to the same diet-treated WT mice, supporting that deletion of Tim-1 promotes HFD-induced liver damage.

Lipocalin-2 [LCN2, also known as neutrophil gelatinase-associated lipocalin (NGAL)] is known to be expressed by a variety of cells including neutrophils, macrophages, epithelial cells like hepatocytes and its upregulation in liver is a reliable indicator of liver inflammation and damage. To further confirm the effects of Tim-1 deficiency on liver injury and inflammation, we examined the mRNA and protein levels of LCN2 in diet-induced fatty livers. As shown in **Figure 5B**, HFD feeding increased hepatic LCN2 mRNA by 34-fold in WT and 83-fold in Tim-1^-/-^ mice (*P* < 0.01 *versus* HFD WT group). Accordingly, Western blot analysis revealed that HFD-induced upregulation of LCN2 protein in liver tissues was substantially enhanced in mice lacking Tim-1 (**Figures 5C, D**). Moreover, Tim-1 deletion accelerated HFD-induced neutrophil infiltration. As shown in **Figures 5C, E**, Ly6G protein was greatly higher in the liver of HFD Tim-1^-/-^ mice compared to HFD WT mice, whereas F4/80 was similarly increased in HFD-fed WT and Tim-1^-/-^ livers (**Figures 5C, F**).

**Figure 6:**
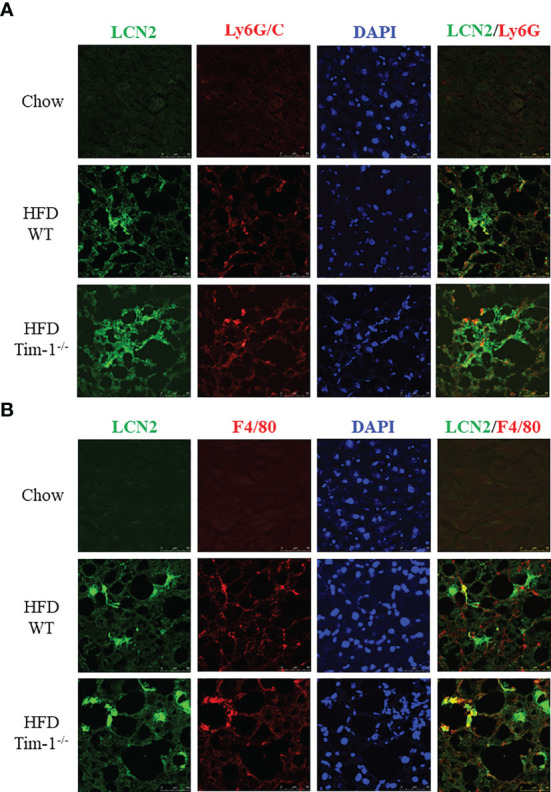
Tim-1 deficiency increased LCN2 expression in infiltrated liver neutrophils and macrophages. Representative immunofluorescence images of double staining for LCN2 with neutrophil marker Ly6G/C **(A)** or macrophage marker F4/80 **(B)** in chow or HFD-fed WT and Tim-1^-/-^ livers.

Next, we determined if neutrophils and/or macrophages are the primary cell sources of elevated LCN2 in the obese liver by dual-labeling of the liver sections for LCN2 with Ly6G or with F4/80. In addition to barely detectable LCN2 staining in normal mouse livers, there were no apparent Ly6G or F4/80 signals in chow-fed WT and Tim-1^-/-^ mouse livers. As depicted in **Figures 6A, B**, increased LCN2, Ly6G, and F4/80 were observed in HFD WT livers, which was further enhanced by Tim-1 deletion. Some but not all LCN2-positive cells were stained positive for Ly6G (**Figure 6A**). Similarly, a subset but not all LNC2-positive cells expressed macrophage marker F4/80. Our results support that Tim-1 deficiency enhanced LCN2 expression in both infiltrated neutrophils and macrophages in HFD mouse livers.

### Tim-1 Deletion Accelerated HFD-Induced Liver Fibrosis

**Figure 7:**
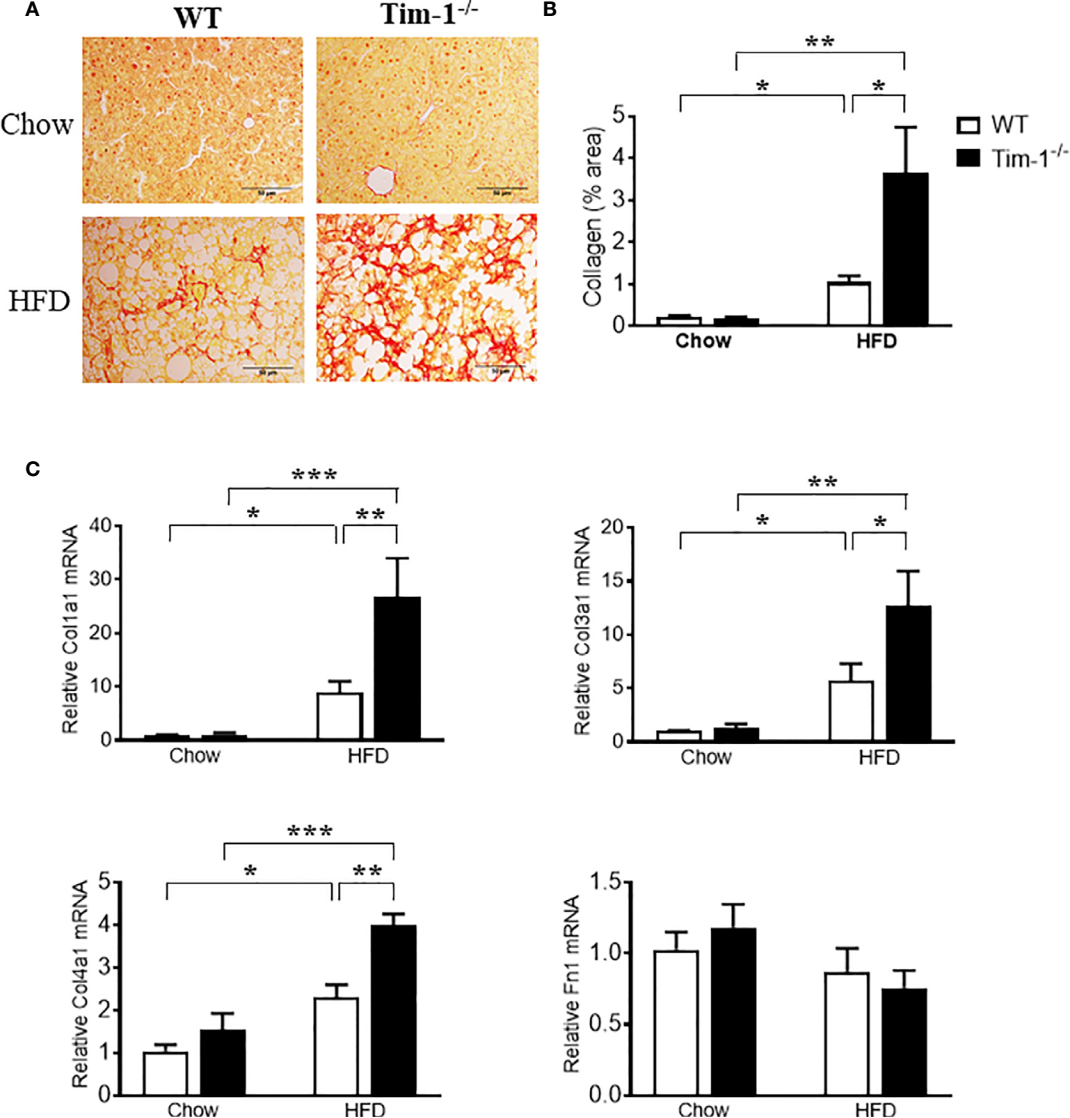
Tim-1 deficiency accelerated HFD-induced liver fibrosis. **(A)** Representative images of Sirius Red staining to identify collagen deposition in liver sections from chow or HFD-fed WT and Tim-1^-/-^ mice. **(B)** Quantitative analysis of Sirius Red-stained liver tissue sections. **(C)** Real-time qPCR analysis of hepatic gene expression of type I (Col1a1), type III (Col3a1), and type IV (Col4a1) collagen as well as fibronectin (Fn1) in chow or HFD-treated WT and Tim-1^-/-^ mice. Values are mean ± SEM. n=4-6 mice. Statistical differences were assessed by one-way ANOVA with Tukey’s multiple comparisons test; *P < 0.05, **P < 0.01, ***P < 0.001.

To further determine if increased hepatic injury and inflammation in HFD Tim-1^-/-^ mice would enhance collagen deposition, we next performed Sirius Red staining on paraffin-embedded liver sections. As depicted in (**Figures 7A, B**), Sirius red staining revealed that HFD feeding for 6 months induced minor to moderate accumulation of collagen fibers in liver tissue of WT mice. Tim-1 deficiency greatly enhanced HFD-induced fibrosis, as shown by abundant fibrillar collagen deposition in HFD Tim-1^-/-^ livers. The liver expresses type I (Col1), III (Col3) and IV (Col4) collagens with type I being the major collagen associated with hepatic fibrosis in Western diet-fed mice and humans with NASH. Compared to chow controls, we found that HFD substantially increased the expression of Col1a1, Col3a1, and Col4a1 by 24-, 10-, and 2.6-fold, respectively in Tim-1^-/-^ livers and 7-, 4-, and 2-fold, respectively in WT livers (**Figure 7C**), while fibronectin 1 (Fn1) transcript levels were not different among the groups.

### Tim-1 Deletion Enhanced M2 Macrophage Polarization

**Figure 8:**
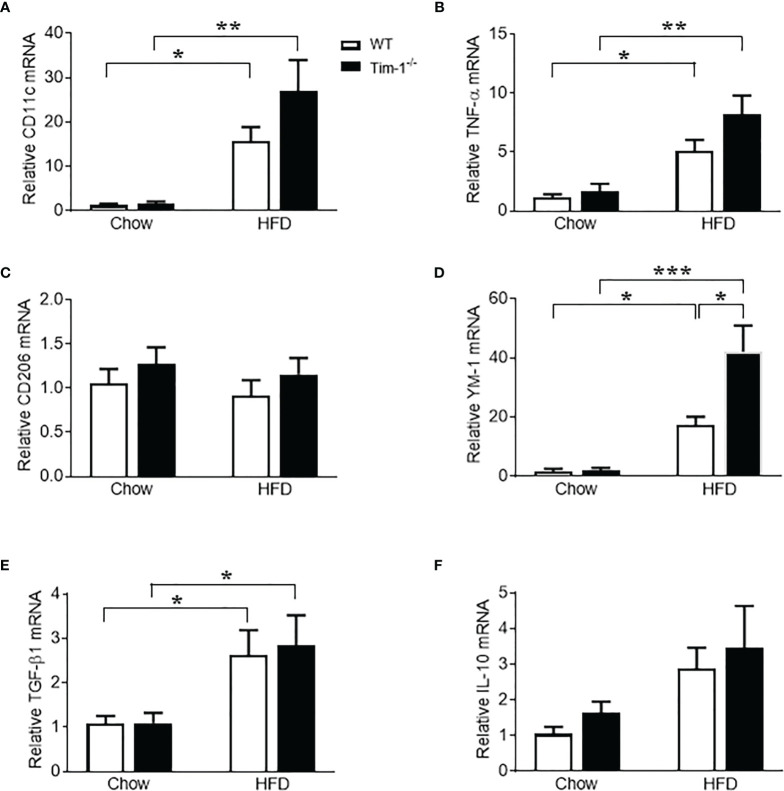
Effects of Tim-1 deficiency on M1 and M2 macrophages. Real-time PCR analysis of hepatic gene expression of M1 macrophage marker CD11c **(A)**, proinflammatory cytokine TNF-α **(B)**, M2 markers CD206 **(C)** and YM-1 **(D)**, and anti-inflammatory cytokine TGF-β1 **(E)** and IL-10 **(F)**. Values are mean ± SEM. n=4-6 mice. Statistical differences were assessed by one-way ANOVA with Tukey’s multiple comparisons test; *P<0.05, **P<0.01, ***P<0.001.

Macrophages have been found to both promote liver fibrosis and contribute to its resolution by reprogramming the two different polarization states, pro-inflammatory M1 macrophages (classically activated) and anti-inflammatory M2 macrophages (alternatively activated), which was driven by micro-environmental cues. To further evaluate the effect of Tim-1 deficiency on macrophage activation and polarization, we next determined the expression levels of markers for M1 and M2 macrophages in liver tissues. Compared to chow controls, HFD feeding caused a similar increase in mRNA levels of M1 marker CD11c (**Figure 8A**) and pro-inflammatory cytokine TNF-α (**Figure 8B**) in both WT and Tim-1^-/-^ mice. As indicated in **Figure 8C**, hepatic expression of CD206, a well-known M2 marker for both mouse and human, was not affected by HFD diet or Tim-1 deletion. In contrast, HFD feeding substantially increased hepatic expression of YM-1, a specific mouse M2 marker, by 18-fold in WT and 42-fold in Tim-1^-/-^ mice (*P* < 0.05 vs. HFD-WT) compared with chow controls (**Figure 8D**). mRNA levels of anti-inflammatory cytokine TGF-β1 were elevated to a similar extent in HFD-fed WT and Tim-1^-/-^ mice (**Figure 8E**). Additionally, hepatic expression of IL-10 was not significantly modified by diet or Tim-1 deletion (**Figure 8F**).

## Discussion

This is the first study that documents the functional significance of Tim-1 in diet-induced NAFLD/NASH. We found that Tim-1 expression was significantly upregulated in the liver of HFD-fed WT mice. Tim-1 deficiency resulted in an exacerbation of HFD-induced hepatic steatosis and inflammation, as evidenced by increased hepatocyte ballooning and excessive lipid accumulation concomitant with augmented infiltration of inflammatory cells as well as pronounced hepatic expression of LCN2 in HFD Tim-1^-/-^ mice. Moreover, HFD-induced collagen deposition and hepatic expression of fibrotic and M2 macrophage markers were significantly enhanced by genetic deletion of Tim-1. Our results suggest that Tim-1 functions in pathways that suppress the recruitment and activation of inflammatory cells in the liver and protect against NASH progression.

In agreement with previous reports (30–35), we found that HFD-fed C57BL/6J mice gained excessive weight and developed NAFLD/NASH as assessed by liver histology within 6 months of HFD feeding. Interestingly, diet-induced liver steatosis was significantly exacerbated by Tim-1 deletion, as evidenced by higher liver weight, more severe hepatocyte ballooning and excessive lipid accumulation in HFD Tim-1^-/-^ mice. These results support a functional role for Tim-1 in lipid metabolism and/or hepatic distribution in response to metabolic stress.

Hepatic steatosis develops when lipid uptake and *de novo* synthesis surpass lipid oxidation and export. In NAFLD, hepatic FA uptake and *de novo* lipogenesis are increased. Long-chain FAs can be either transported directly by FATPs across the plasma membrane or, alternatively, are first accumulated on the plasma membrane by binding to CD36, which subsequently transfers FAs to transport proteins. As a member of glycoprotein, FA translocation enzyme CD36 is weakly expressed in hepatocytes and liver tissue under physiological conditions but significantly upregulated in animal models and NAFLD patients (36, 37). Moreover, previous studies confirmed that overexpression of CD36 led to steatosis in mice, and liver-specific knockout of CD36 reduced lipid content in mice fed with HFD (23, 38). Our finding that Tim-1 deletion greatly enhanced HFD-induced upregulation of hepatic CD36 protein expression suggests that Tim-1 deficiency may disrupt lipid homeostasis by increasing FA uptake *via* its modulation of CD36. In addition, hepatic expression of SREBP1 protein, an important transcriptional promoter of lipogenesis activated by insulin signaling in the fed state, was induced by HFD treatment in WT mice, which was further upregulated by Tim-1 deletion. Together, our results strongly support that Tim-1 deficiency may accelerate the severity of hepatic steatosis by increasing both FA uptake and *de novo* lipogenesis.

Next, we confirmed that Tim-1 deficiency aggravated HFD-induced liver injury and inflammation, as evidenced by elevated serum ALT, and increased LCN2 mRNA and protein in liver tissues. One of the striking effects of Tim-1 deficiency in diet-induced NAFLD was substantially increased hepatic expression of LCN2 concomitant with increased infiltration of inflammatory cells including neutrophils and macrophages. Moreover, confocal immunofluorescence images revealed a partial colocalization of LCN2 with neutrophil marker Ly6G or with macrophage marker F4/80, demonstrating both neutrophils and macrophages as the major cell populations contributing to Tim-1-enhanced LCN2 upregulation in diet-induced NAFLD/NASH mouse model. LCN2, an acute protein induced in response to bacterial infection, metabolic stress, or injury, has been proven to be a reliable biomarker of liver injury and inflammation. Previous studies also demonstrated that injury-induced upregulation of hepatic LCN2 has a significant hepatoprotective effect in acute liver injury and that hepatocytes are the major source for hepatic LCN2 (39–41). Furthermore, these data suggest that LCN2 might act as an intrinsic “help-me” sensor upon injury to recruit inflammatory cells. In line with this assumption, Asimakopoulou et al. observed that Lcn2-deficient mice showed a significantly lower recruitment of neutrophils and leukocytes, compared to WT animals, when fed with a methionine-choline-deficient (MCD) diet that induces hepatic inflammation and injury (42). A recent report also provides evidence that LCN2 is implicated in the progression of simple steatosis to NASH by promoting neutrophil-macrophage crosstalk (43). The authors found that Lcn2-deficient mice on high-fat high-cholesterol (HFHC) diet had reduced infiltration of both neutrophils and macrophages, and chronic LCN2 administration-induced elevation of hepatic macrophages was abrogated by Ly6G antibody-mediated depletion of neutrophils (43). Likewise, mice treated with ethanol exhibited elevated LCN2 expression in neutrophils, and Lcn2-deficient mice are protected from alcoholic steatohepatitis (ASH) as demonstrated by reduced neutrophil infiltration and liver injury (44). Thus, Tim-1 deficiency may accelerate obesity-related liver inflammation and injury by upregulating LCN2, which warrants further investigation.

Upon chronic inflammation and injury, hepatic stellate cells (HSCs) are activated and turn into the primary source of extracellular matrix in ASH and NASH. A recent study provides evidence that hepatic LCN2 is also involved in the activation of HSCs in ASH (45). The authors found that hepatic expression of Col1a1 was elevated in ASH patients and correlated with hepatic LCN2 expression, and Lcn2-deficient mice were protected from liver fibrosis caused by either ethanol or CCl4 exposure. The causal role of LCN2 in tissue fibrosis is also supported by an *in vitro* study that recombinant LCN2 induced type 1 collagen protein expression in human fibroblasts in a dose-dependent fashion (46). In the current study, we found that mice lacking Tim-1 showed an enhanced fibrogenic response associated with an upregulation of LCN2 expression in the liver of HFD-fed mice.

The development of fibrosing steatohepatitis is a complex process that involves multicellular responses other than HSCs. For example, activated macrophages can differentiate into diverse phenotypes contributing to both the progression and regression of the fibrotic process (47–49). Deletion of the macrophage population either during injury or during repair/resolution has dramatically different effects on the overall fibrotic response (49, 50). Specifically, macrophage depletion in progressive inflammatory injury results in amelioration of fibrosis; in contrast, depletion during recovery results in a failure of resolution with the persistence of cellular and matrix components of the fibrotic response (49, 50). Using a set of established markers, Belijaars et al. further localized and quantified M1 (classically activated)- and M2 (alternatively activated)-dominant macrophages in CCl4-damaged mouse livers as well as human end-stage cirrhotic livers (47). They found that M2 markers were present in liver fibrotic lesions but nearly absent during the resolution of fibrosis, suggesting a more pro-fibrotic character of M2-dominant macrophages in human and mouse livers (47). In this study, we also quantified hepatic gene expression of M1 and M2 markers to further determine if Tim-1 is involved in macrophage polarization and activation, A similar upregulation of CD11c and TNF-α gene expression was observed in HFD-fed WT and Tim-1^-/-^ livers, suggesting that Tim-1 may not be required for M1 macrophage polarization. We next determined CD206 (mannose receptor, MCR-1) and YM-1, M2 macrophage markers, in mouse NAFLD/NASH livers. Hepatic mRNA levels of CD206, a well-known marker for both mouse and human M2 macrophages, was unaltered by either HFD diet or Tim-1 deletion. While in many organs M2 macrophages specifically express CD206, in livers its expression was found in macrophages as well as in sinusoidal endothelial cells (47), which complicate the quantitative interpretation of mRNA expression analysis of whole liver homogenates. Of note, YM-1 does not have this disadvantage and acts as a specific and useful marker for M2 macrophages in mouse liver (47, 51). Therefore, a substantial enhancement of HFD-induced hepatic expression of YM-1 transcript by loss of Tim-1 supports an important role for Tim-1 in M2 macrophage polarization.

Previous studies have demonstrated that M2-dominant macrophages, activated by Th2 cytokines (e.g., IL-4 and IL-13), are associated with increased fibrogenesis, tissue remodeling, and angiogenesis (52–54). When cultured with myofibroblasts, M2 macrophages promote complex matrix deposition (52, 53, 55, 56). Although Tim-1 has been proposed to have both activating and inhibitory effects in immune responses by studies using different monoclonal antibodies (8–15), a recent study performed in *in vivo* allergic airway disease revealed enhanced inflammatory responses and production of the Th2 cytokines IL-4, IL-5, and IL-13 in the absence of Tim-1, suggesting that its primary role is to dampen, rather than promote, Th2-type immune responses (16). Here, we found that Tim-1 deficiency enhanced HFD-induced macrophage accumulation and M2 polarization concomitant with accelerated fibrotic response in the mouse NAFLD/NASH model. It is likely that Tim-1 exerts an anti-fibrotic effect by inhibiting alternative activation of macrophages though its modulation of Th2-type immune response.

Our results suggest that Tim-1 deficiency may accelerate HFD-induced fibrosis by increasing macrophage infiltration and M2 polarization. However, we cannot answer where the M2-dominant macrophages come from, meaning are they derived from bone marrow monocytes, or do they develop from tissue-resident Kupffer cells. Previous studies showed that monocytes do infiltrate the liver during fibrogenesis and resolution. Moreover, during sustained Th2-type profiles, alternatively-activated Kupffer cells may be essential contributors in collagen synthesis, probably leading to an active fibrogenic state (53, 57, 58). Therefore, understanding the dynamics of all these different macrophages during fibrogenesis as well as the interactions between immune cells and macrophages is a subject of future research interest.

We are also aware that the use of global knockout mice, to define the role of Tim-1 in NAFLD/NASH, does not allow to distinguish between its direct hepatic and extrahepatic effects. It is well known that Tim-1/Kim-1 is massively induced in damaged renal proximal tubules after acute and chronic kidney injury (59, 60). Moreover, Tim-1/Kim-1 expression is anti-inflammatory and reduces acute kidney injury due to its mediation of phagocytic processes in renal tubular cells (59, 60). There is growing evidence that NAFLD and chronic kidney disease share common pathogenetic mechanisms and that the fatty liver per se may promote kidney injury and *vice versa* (61). In the present study, we used the C57BL/6J strain, one of the most susceptible to obesity but relatively resistant to kidney injury when fed high fat diets (62). Of note, we found that HFD feeding did not alter Tim-1/Kim-1 expression in the kidney of WT mice, suggesting that renal Tim-1 may play a minor role in diet-induced NAFLD/NASH. To better define the role of hepatic Tim-1, however, using tissue-specific Tim-1 knockout mouse model is warranted in the future.

In summary, this study revealed that Tim-1 deficiency led to an enhancement of lipid accumulation, and liver inflammation and injury in diet-induced NAFLD/NASH mouse model. In addition, we provide evidence that Tim-1 expression regulates lipid metabolism in hepatocytes by targeting CD36 and SREBP1. Our results indicate a regulatory role for Tim-1 in HFD-induced steatohepatitis and might be considered as a target for the prevention and treatment of chronic liver disease.

## Data Availability Statement

The raw data supporting the conclusions of this article will be made available by the authors, without undue reservation.

## Ethics Statement

The animal study was reviewed and approved by Institutional Animal Care and Use Committee at the Morehouse School of Medicine.

## Author Contributions

JG and YZ have contributed equally to this work and share first authorship. JG, YZ, and XZ designed, performed and analyzed data from most of the experiments. JS and JMS performed histology and Western blot analysis. XZ conceived and supervised all studies. XZ wrote the manuscript. JI reviewed and edited the manuscript. All authors contributed to the article and approved the submitted version.

## Funding

This work was supported by the National Institutes of Health Grants SC1DK112151 and S21MD000101.

## Conflict of Interest

The authors declare that the research was conducted in the absence of any commercial or financial relationships that could be construed as a potential conflict of interest.

## Publisher’s Note

All claims expressed in this article are solely those of the authors and do not necessarily represent those of their affiliated organizations, or those of the publisher, the editors and the reviewers. Any product that may be evaluated in this article, or claim that may be made by its manufacturer, is not guaranteed or endorsed by the publisher.
